# Stem cell therapy in ophthalmology: emerging clinical trials and regulatory developments

**DOI:** 10.1097/JS9.0000000000002377

**Published:** 2025-04-03

**Authors:** Youran Cai, Qing Zhou

**Affiliations:** Department of Ophthalmology, the First Affiliated Hospital of Jinan University, Guangzhou, China

Stem cell therapy has emerged as a revolutionary approach in regenerative medicine, and ophthalmology is one of the first medical science branches to benefit from it. Potential treatments for incurable ocular diseases such as age-related macular degeneration (AMD), retinitis pigmentosa (RP), and limbal stem cell deficiency (LSCD) are offered by stem cell-based therapy^[^1^]^. In recent years, stem cell therapy in ophthalmic diseases has reached remarkable progress. While current standard treatments including intraocular pressure-modulating pharmacotherapy or anti-VEGF therapies, address symptoms but fail to restore lost cells or reverse disease progression, underscoring unmet clinical needs.

Stem cell-based strategies aim to regenerate damaged tissues by retinal ganglion cells or retinal pigment epithelium (RPE), leveraging the pluripotency of embryonic stem cells (ESCs), induced pluripotent stem cells (iPSCs), or mesenchymal stromal cells (MSCs)^[^2^]^. Clinical translation remains challenging despite promising preclinical results. Key barriers include heterogeneity in trial designs, cell sources, and safety concerns. Our research provides a comprehensive landscape analysis of ongoing and completed clinical trials and evaluates and integrates recent preclinical advancements to guide future research.

We conducted systemic research on the Trialtrove database for clinical trials on stem cell therapies for ophthalmology diseases, limited to 31 December 2024. After a thorough title review, 11 trials were excluded as the tested drugs were noncompliant with stem cell therapies. A total of 88 clinical trials of stem cell therapies for ophthalmology diseases were enrolled in the final analysis. We investigated the trends of number, phases, disease, cell types, and target conditions of all the selected clinical trials to evaluate the effectiveness of stem cell therapies for different ophthalmic diseases.

From 2006 to 2024, the number of clinical trials shows a fluctuating upward trend, and peaks in 2011-2015 and 2018-2019 (Fig. 1A). Phase I/II accounted for the highest proportion (34, 38.64%), followed by Phase I and Phase II (Fig. 1B). The early stage remains most of the clinical phases showing a growing interest in and commitment to exploring new stem cell therapies during this period. Although most clinical trials remained completed, the paucity of later stages reveals a critical translational gap. Multiple measures should be emphasized to promote conversion, such as including technology innovation and regulatory coordination.

**Figure 1.**
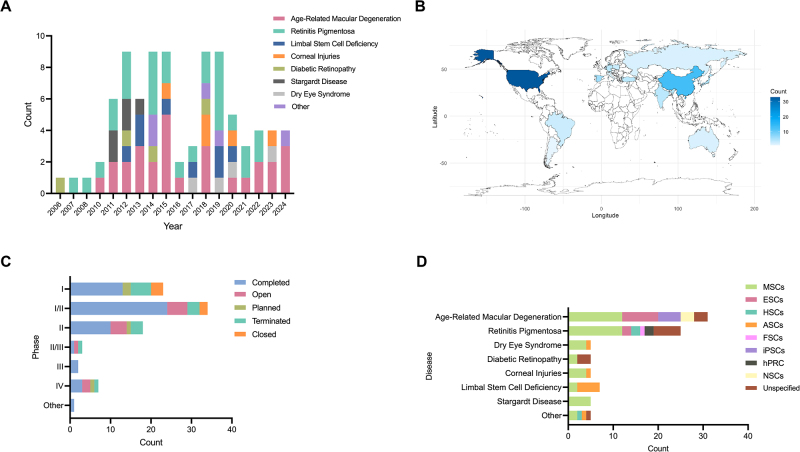
A. The trend of clinical trials worldwide from 2006 to 2024, with each including the total number of trials and a breakdown by disease. B. Global distribution of stem cell therapy clinical trials in ophthalmology. C. The number of clinical trials of different phases, detailing the total number of trials for each phase along with a breakdown by their respective statuses. D. Stem cell therapies for all types of ophthalmology diseases are categorized by the specific stem cell types used. MSCs, mesenchymal stem cells; ESCs, embryonic stem cells; HSCs, hematopoietic stem cells; ASCs, adult stem cells; iPSC, induced pluripotent stem cells; hPRC, human progenitor retinal cells; NSCs, neural stem cells.

All the clinical trials were spread over 20 countries, with a dominating in the United States (33, 37.50%), followed by China (13, 14.77%) and the United Kingdom (8, 9.09%) (Fig. 1C). Over half of the countries are from the European region, and five are Asian countries (Table S1, http://links.lww.com/JS9/E50). The non-country is from Africa, representing disparities in study capacity and participation across different regions. mesenchymal stem cells (MSCs) therapies occupied the majority of all stem cell therapies (43, 48.86%), and the second most popular was ESCs (Fig. 1D). MSCs remain the main stem cell therapy due to their low immunogenicity, multipotent differentiation capacity, and ethical accessibility.

Most of the clinical trials contributed to retinal diseases, exactly AMD and RP (Fig. 1D). The predominance of the two disease reflects their high unmet medical need and well-characterized pathophysiology. Although RP accounted for the second most, the number of RP clinical trials decreased even to zero in recent years. This variation may be associated with its high genetic heterogeneity, with more than 100 genes discovered^[^3^]^. In addition, the pathology involves multilayered structures of the retina limits the efficacy of a single stem cell therapy. CD34+ cell therapy for vision loss of RP has completed a Phase I clinical trial recently (NCT04925687)^[^4^]^. Hematopoietic stem cells (HSCs) therapy appears to be a potential treatment for improved RP vision. Concerning RP clinical trials in the future, there might be more development of genetically modified stem cells or complex therapies combined with optogenetics.

Regarding AMD, the trend of numbers is almost stable and continues to increase from 2020-2024. Several stem cell therapies including MSCs, ESC-derivatives, and iPSC-derivatives are applied on AMD with both industry and academy contributions. Occupation of derivatives has aroused interest worldwide in Geographic Atrophy (GA) of dry AMD treatment in recent years, as GA is considered to be an uncured disease. Indian industry conducted a Phase I/IIa dose-escalation study of Eyecyte-RPE on Geographic Atrophy (GA) of dry AMD patients (NCT06394232). Another iPSC-derived RPE therapy named HLCR-011 has been planned to conduct a Phase I clinical trial since 2022 after being terminated in 2014. In addition, ESC is another stem cell treatment that raised worldwide concern. OpRegen (NCT05626114) and RPESC-RPE-4W (NCT04627428) are both ESC derivatives used to control GA^[^5^]^. The recorded iPSC derivatives clinical trials are targeted to Asians, while ESCs only was conducted in the United States, different types of stem cell therapy should be used in more different regions to make these therapies widely applicable.

**HIGHLIGHT**

- Clinical trials in stem cell therapy for ophthalmology are predominantly early-phase, emphasizing innovation but lagging in late-stage translation.
- Geographically, the U.S., China, and Europe lead 88 trials, with no African participation, highlighting regional disparities.
- Mesenchymal stem cells (MSCs) dominate while iPSC/ESC therapies target geographic atrophy in AMD.
- Retinal diseases drive research (70% focus on AMD and RP), though RP trials decline due to genetic complexity, prompting exploration of novel combinatorial or gene-modified approaches.

In conclusion, stem cells occupied in ophthalmology continue to be a popular tissue worldwide over the years. Most clinical trials were intended on retina disease exactly RP and AMD. In addition, early-status clinical trials are the main type phases, suggesting that potential new stem cell therapies will benefit ophthalmology diseases. With the rapid progress of stem cell interventions, rigorous clinical trial processes, appropriate regulation, and post-market surveillance to ensure the effectiveness and safety of stem cell products should be highlighted.

## Data Availability

All data for this study have been provided.
